# Application of 3D Printing-Assisted Articulating Spacer in Two-Stage Revision Surgery for Periprosthetic Infection after Total Knee Arthroplasty: A Retrospective Observational Study

**DOI:** 10.1155/2021/3948638

**Published:** 2021-02-08

**Authors:** Lingtong Kong, Jiawei Mei, Wufei Ge, Xiansheng Jin, Xiaoxuan Chen, Xianzuo Zhang, Chen Zhu

**Affiliations:** ^1^Department of Orthopedics, The Affiliated Provincial Hospital of Anhui Medical University, Hefei 230001, China; ^2^Department of Orthopedics, The First Affiliated Hospital of USTC, Division of Life Sciences and Medicine, University of Science and Technology of China, Hefei 230022, China; ^3^IAT-Chungu Joint Laboratory for Additive Manufacturing Anhui Chungu 3D Printing Institute of Intelligent Equipment and Industrial Technology, Wuhu 241200, China; ^4^College of Chemistry and Chemical Engineering, Xiamen University, Xiamen 361005, China

## Abstract

**Background:**

Bone cement spacers are widely used in two-stage revision surgeries for periprosthetic joint infection (PJI) after total knee arthroplasty. Current spacer design results in insufficient release of drugs; therefore, current spacers have low efficacy. In this study, we explored a set of alternative articular spacer using 3D printing technology. This novel spacer will increase effectiveness of revision surgery for PJI.

**Methods:**

The spacer was designed using CAD software and constructed on site using 3D-printed silicone mold during debridement surgery. We carried out a retrospective study among patients undergoing treatment using traditional static and new articular spacers. Infection control rate, bone loss, difficulty of revision surgery, knee joint range of motion, function evaluation, and subjective satisfaction of the patients in the two groups were compared.

**Results:**

Forty-two patients undergoing knee revision surgery between Jan 2014 and Nov 2019 were included in this study. Twenty-two patients were treated with static antibiotic cement spacers, whereas the other twenty patients were with treated with 3D printing-assisted antibiotic loaded articulating spacers. Patients in the articular group showed significantly lower bone loss on the femur site and tibial site compared with patients in the static group. In addition, patients in the articular group showed significantly less operation time, intraoperative blood loss, and improved knee function and patient overall satisfaction compared with patients in the static group.

**Conclusions:**

The 3D printing-assisted articular spacer provides satisfactory range of motion during the interim period, prevents bone loss, facilitates second-stage reimplantation and postoperative rehabilitation, and results in low reinfection and complication rates.

## 1. Introduction

Total knee arthroplasty (TKA) is an effective method for treatment of end-stage knee disorders, including osteoarthritis, traumatic arthritis, and inflammatory arthritis [1, 2]. Approximately, 400,000 TKA procedures are performed in the United States yearly [3]. However, TKA surgery is associated with periprosthetic joint infection (PJI). Primary TKA is associated with approximately 0.5%-2.0% PJI incidence, whereas total knee revision (TKR) surgeries are associated with approximately 15%-20% PJI incidence [4–6].

Although new approaches for treatment of PJIs such as debridement and implant retention (DAIR) and one-stage revision have been proposed, two-stage revision strategy remains the gold standard treatment for chronic PJI after TKA [7–9]. Previous studies report that the infection control rate after treatment using two-stage revision strategy is approximately 80%-90% [9, 10]. The patient undergoes at least two surgeries in the two-stage revision procedure. The first surgery is carried out to remove original prosthesis, debride the knee joint, and insert an antibiotic cement spacer to control infection. The spacer can be replaced with a revision prothesis after inflammation elimination and infection control.

Different temporary spacers are used in clinical practice. In the temporary spacers, the surgeon first fills the joint cavity with unshaped bone cement which is solidified during surgery. The filling allows the knee to be fixed in a functional position without leaving dead space thus minimizes infection [11]. Articular spacer is used to avoid joint stiffness [12]. Hsu et al. reports a bone cement spacer mold that simplifies and standardizes the spacer fabrication process [13]. In addition, alternative approaches such as use of partial original prothesis [14], metal spacers [15], and various commercially available products available for clinical application have been reported [16, 17].

Cement spacers are mainly prepared on table during surgery. Currently, there is no consensus on an optimal spacer. Spacer choices mainly depend on preference of the surgeon, balance between hand manufactural convenience, size matching, and functional performance. Most temporary bone cement spacers lack mechanical considerations unlike mature artificial joint prostheses; therefore, they are not conducive for knee functions. This may be an overlook in intraoperative manufactural instructions. Predesigned molds for spacer casting are short in size and models and unable to adapt to severe conditions such as bone defects and ligament insufficiency during revision surgery.

Recent advances in technology have resulted in development of 3D printing technology. Recently, applications of 3D printing technology in orthopaedics have steadily increased. 3D printing enables rapid prototyping based on customs of individual patient, thus improving anatomic matching and mechanical stability. In this study, we designed a complete set of solutions based on rapid prototyping and 3D printing. Further, we used the 3D-assisted solutions to construct antibiotic-loaded bone cement spacer holders for use in two-stage knee revision of PJI. We hypothesized that this 3D printing-assisted solution will facilitate knee revision surgery and provide better clinical effects. We then compared the preliminary outcomes after using 3D-assisted antibiotic-loaded spacers TKR in this study with outcomes of static bone cement spacers obtained in previous studies.

## 2. Materials and Methods

### 2.1. Design and Preparation of the Articular Spacer

**Figure 1:**
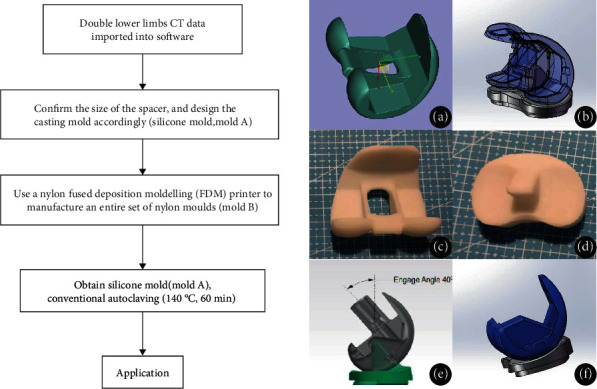
The production process and design of 3D printing assisted articulating spacer. (a) The spacer imitates the geometry of a permanent artificial knee joint, and the anterior condyle is thinned to reduce anterior patellar pressure. (b) The diseased carriage is extended, deepened, and lengthened, enabling the patella to enter the carriage movement early. Therefore, patients are less likely to have adverse reactions such as dislocation and ringing after surgery. (c, d) Femoral component and tibial component of the 3D printing-assisted articulating spacer. (e) The tibial column is elevated to provide effective posterior stability. (f) The tibial platform sliding interface is designed with a deep disc, allowing a range of motion for joint stability.

A prototype of the bone cement spacer (Figure 1) was designed using CAD software (OpenSCAD v2013.01, http://www.openscad.org/). The spacer consisted of a femoral component and a tibial component imitating the geometry of a permanent artificial knee joint. Femoral condyles were shaped on both sides to effectively reduce pressure on the soft tissue. The anterior condyle was thin to reduce anterior patellar pressure and avoid anterior patellofemoral pain. The diseased carriage was extended, deepened, and lengthened, to help the patella enter the carriage movement early. Therefore, it reduces the risk of adverse reactions such as dislocation and ringing after surgery. Tibial side of the AP/ML ratio was designed with reference to anatomical characteristics of the Asian race to provide better platform coverage. Thickness of the tibial platform component was adjustable according to the platform bone deficit, and the tibial platform was tilted 5° posteriorly to provide effective anterior stability. The tibial column was elevated to provide effective posterior stability. Further, the tibial platform sliding interface was designed with a deep disc, sacrificing some range of motion for a joint stability, reducing ligament wear during absenteeism. The platform column was designed to match the intercondylar fossa, allowing for restriction of the occupying apparatus, thus providing lateral stability.

The production process of 3D printing-assisted articulating spacer was as follows. First, CT images of the contralateral knee of the patient were obtained and calculated to confirm the size of the spacer and for designing the casting mold accurately (silicone mold, mold A). The corresponding CAD model was selected from the digital library and resized based on actual needs, and the file was exported in STL format. An entire set of nylon molds (mold B) was then manufactured using a nylon fused deposition modelling (FDM) printer. The silicone solution and curing agent were mixed in a 1 : 1 mass ratio, stirred under vacuum (100 pa) to remove air bubbles. The solution was poured into the prepared nylon mold (mold B) for casting. The workable time of silica gel is about 20 minutes (at 25°C room temperature); therefore, silica gel should be injected into the nylon mold immediately after removing air bubbles. Stirring of the solution in vacuum should be repeated to remove bubbles if necessary. The silicone was cured in a constant temperature heating oven at 60°C for about 8 hours (increasing the temperature could reduce curing time; however, high temperatures accelerate aging of the silicone). The silicone mold (mold A) was then sterilized by conventional autoclaving (140°C, 60 min) and used in surgery.

Bone cement spacer was prepared on site during surgery. PMMA solution and fluoroscopically photographic crosslink catalyst powder (AGC Style Company Biomet Orthopaedics Inc., Warsaw, USA) were thoroughly mixed, injected, and pressed into the silicone molds with special tools. The composite was placed at room temperature for 8-10 minutes to obtain the complete bone cement spacer. Spacer installation was done during surgery.

### 2.2. Clinical Data Acquirement

**Figure 2:**
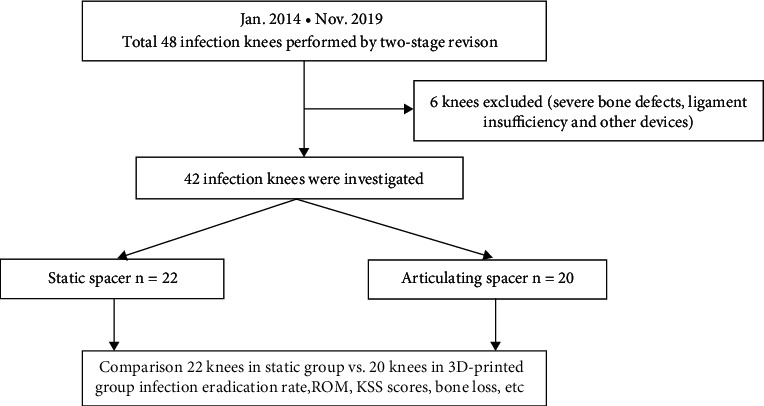
Flow chart on the design of this retrospective observational study.

A retrospective study was conducted after obtaining approval from IRB. The experimental design of this study is shown in Figure 2. We performed a chart review using the medical record system. Patients receiving total knee revision surgeries between Jan 2014 and Nov 2019 were included in this study. The inclusion criteria were (1) patients clinically or pathogenically diagnosed with PJI after TKA, (2) patients who underwent 2-stage procedures involving use of an interim antibiotic bone cement spacer, and (3) patients treated with hand-made static spacer or 3D printing assistant articular spacer. The exclusion criteria included (1) patients who received other surgical treatments before (e.g., DAIR and amputation), (2) patients with severe bone defects (>30 mm) and ligament insufficiency before revision surgery, (3) patients who used other devices as interim spacers (e.g., femoral component in hip prothesis) before the surgery, and (4) patients who failed to complete a 6-month follow-up. Diagnosis of PJI was carried out according to recommendations by International Consensus Meeting on Peri-prosthetic Joint Infection (ICMPJI) in 2013 [18]. Two reviewers independently screened and decided on inclusion of cases. A third senior doctor made the final decision in case of disagreement.

Patient baseline characteristics, including age, gender, primary disease comorbidities, and pathogenic microorganisms were retrieved from the admission assessment form in the medical records. Interval time between two operations, reinfection rates, required tibial tubercle osteotomy in revision surgery, and operation time in second-stage surgery was obtained by searching linked medical records using the unified health record management system. Radiologic images before and after surgery and images obtained from follow-ups were acquired from the hospital picture archiving and communication system (PACS). Bone loss was defined as the distance between original joint line and current remained bone substance. Bone loss was observed and calculated on anteroposterior and lateral X-ray image. Range of motion was measured before discharge from the hospital. Knee flexion deformity exceeding 20° or joint range of motion less than 45° was considered a serious joint mobility disorder. The KSS score system was used to evaluate the patient knee function [19]. The Danish Health and Medicine Authority questionnaire was used for satisfactory evaluation [20]. An overall satisfactory score above 7 showed that the patient was satisfied with the treatment process. Data for questionnaires were obtained during clinical follow-ups or through telephone calls.

### 2.3. Hospitalization Management

All patients were hospitalized in general wards or infectious units in our joint centre. Preoperative evaluation, blood tests, and pathogen detection were performed after admission. Surgical treatments were divided into two stages. In the first stage, original infection-related prosthesis and bone cement were removed and cultured. Antibiotic loaded bone cement was used as the controlled release system, either through static or articular approach. Patients were given intravenous or oral drugs against infections after debridement surgery until infectious indicators, and bacterial culture was all negative. Patients were then admitted for revision surgery; otherwise, another debridement was performed. Patients received standard rehabilitation schedules between two surgeries and after the final revision.

### 2.4. Surgical Techniques

**Figure 3:**
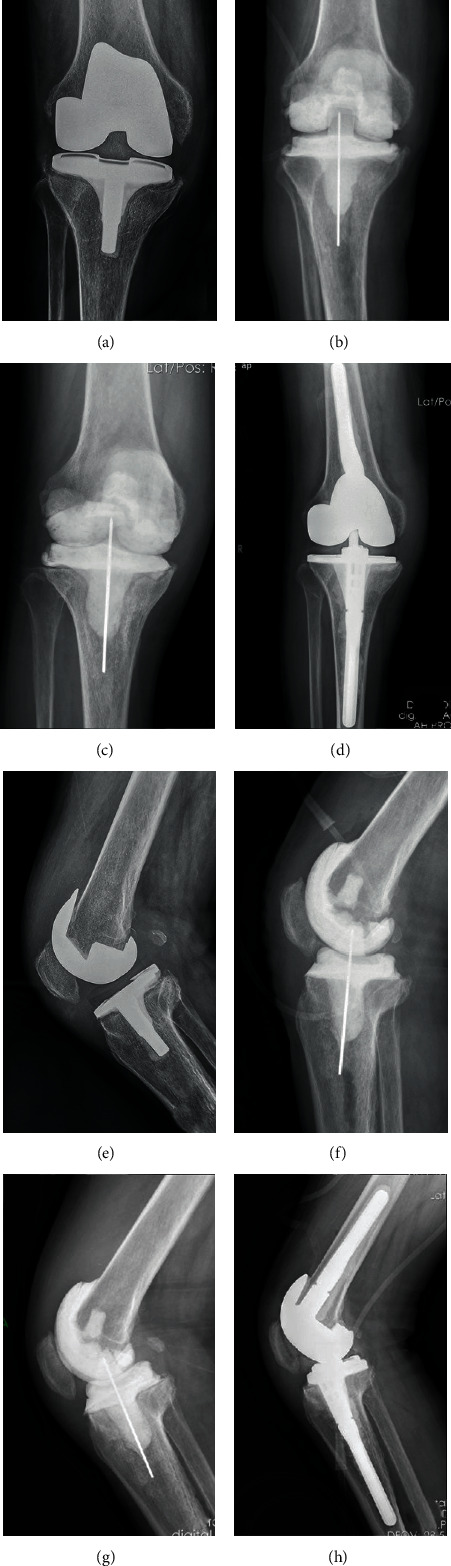
(a, e) X-ray films of periprosthetic joint infections. (b, f) X-ray films immediately after insertion of 3D printing-assisted articulating spacer. (c, g) Eight weeks later, X-ray films showed no obvious bone loss. (d, h) X-ray films immediately after the second-stage revision.

**Figure 4:**
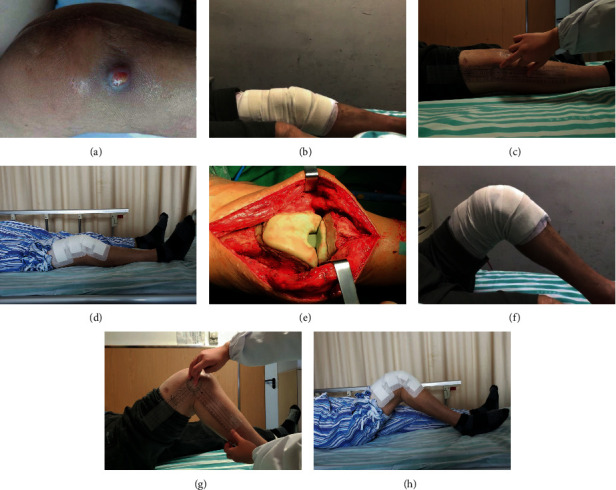
(a) Periprosthetic infection after TKA, abscess is about to break. (e) Installation of 3D printing-assisted articulating spacer. (b, f) The knee joint is straightened and knee flexion is about 85° after spacer insertion. (c, g) Eight weeks later, the knee flexion is about 90°. (d, h) Flexion and straightening function of the knee joint was good after the second stage revision.

Debridement and revision surgeries were performed using original incision. Original prothesis and bone cements were removed in the 1st stage debridement. Iterative irrigation was performed using 1% iodophor solution, 3% hydrogen peroxide solution, and normal saline after thorough debridement. Antibiotic bone cement was formulated, plasticized, and installed into the cavity of the joint. The antibiotic was prescribed following preoperative pathogen culture and drug-sensitivity test. A broad-spectrum coverage of antibiotic scheme was used for clinically confirmed PJI with negative culture [21]. A mixed formulation containing 1 g vancomycin, 0.8 g meropenem, 0.4 g fluconazole, and 0.3 g isoniazid in every 40 g Heraeus bone cement was used. A silicone tube was placed in the joint cavity for continuous drainage until an effusion less than 50 ml per day was achieved. Static spacer was used to fill the joint cavity between the femur and tibia. 3D printing assistant articular spacer was casted on table using sterile silicone molds as described before. Tibial and femoral component was installed separately using additional adhesive cement. Details on patients who received articular spacer are shown in Figures 3 and 4.

A 2nd revision surgery was performed after the infection was completely controlled. The bone cement spacer was replaced with revision joint replacement prostheses. Proper knee alignment adjustment and soft tissue balancing were performed during the surgery. Cavernous and inclusive bone defects were filled with bone cement, and no-inclusive or structural bone defects were repaired using renovation pads. Tibial tuberosity osteotomy (TTO) was performed for stiff knees.

### 2.5. Statistical Analysis

Statistical analysis was performed using SPSS, version 20.0 (SPSS Inc., Chicago, Illinois). Normally distributed data were presented as mean ± standard deviation (SD), whereas non-normally distributed data were presented as median ± interquartile range (IQR). The paired *t*-test was used to compare changes in postoperative range of motion in each group. The unpaired *t*-test was used to compare the KSS score, bone loss, second-stage operation time, and intraoperative blood loss between the two groups. The *χ*2 test was used to compare differences in the number of osteotomies, patellar tendon contracture, and reinfection between the two groups.

## 3. Results

### 3.1. Patient Characteristics

A total of 48 PJI cases of patients who received two-stage revision surgery after a comprehensive review of medical records between Jan 2014 and Nov 2019 were included in this study. Six knees were excluded as they showed severe bone defects, ligament insufficiency, and other devices that had been used before. A total of 42 patients with infected knees were included in this study. Out of the 42 cases, 22 patients were treated with static antibiotic cement spacers, whereas the other 20 patients were treated using 3D printing-assisted articulating spacers.

The average age of patients in the static group was 67.2 ± 10.1 years. The mean follow-up period of the static group was 43 months (ranging 30-61 months). The average age of patients in the 3D-printed articulating group was 65.5 ± 11.4 years. The mean follow-up period for the 3D-printed articulating group was 18 months (ranging 8-28 months). The primary conditions for the 42 infected knees were osteoarthritis in 30 knees, rheumatoid arthritis in 11 knees, and ankylosing spondylitis in 1 knee. Details on baseline characteristics are summarized in Table 1.

Infection in most of the subjects was completely controlled after single debridement, whereas two patients (one using static spacer and the other using articular spacer) required secondary debridement and change of spacer. All patients recovered after the treatment cycle.

### 3.2. Pathogen Spectrum

Joint fluid culture showed different sources of bacterial infection. The most common pathogens observed in the cultures were methicillin susceptible *Staphylococcus aureus*, which was found in 16 knees, and *Staphylococcus epidermidis* in 9 knees. A total of 4 knees showed a negative culture. High-throughput sequencing was used to detect the presence of pathogenic bacteria in some cases. Details on pathogen spectrum are shown in Table 2.

### 3.3. Bone Loss

**Figure 5:**
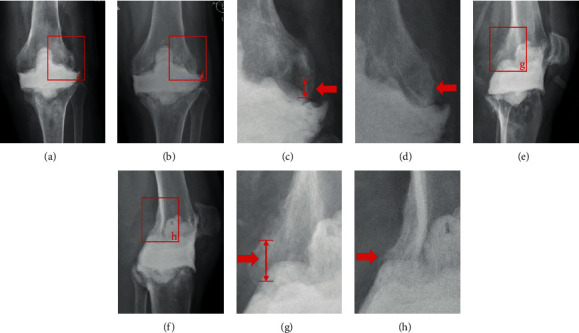
(a, e) X-ray films immediately after static spacer insertion. (b, f) Nine weeks later, X-ray films showed bone loss. The upward displacement of the spacer relative to the original joint line is the bone loss of the femur, and the downward displacement of the spacer relative to the joint line is the bone loss of the tibia. (c) and (d) show the amount of bone loss in the distal femur on the anteroposterior X-ray films. (g) and (h) show the amount of bone loss in the distal femur on lateral X-ray films.

Bone loss was measured using anterior-posterior and lateral X-ray radiographs as shown in Figure 5. Bone defect over 5 mm under joint line was considered as significant bone loss. Among the 42 knees, all 22 with static spacers had significant bone loss, whereas only 4 patients in the articulating group showed bone loss. The mean femoral bone loss in the static group was 9.6 mm (range, 5-20 mm), whereas tibial bone loss in this group was 5.5 mm (range, 0-10 mm). In the 3D-printed articulating group, the mean femur bone loss was 1.7 mm (range, 0-10 mm), whereas mean tibial bone loss was 1 mm (range, 0-5 mm). Bone loss in the articulating group was significantly lower compared with bone loss in the static group (Table 3).

### 3.4. Second-Stage Revision

All patients received secondary revision surgery. The average operation time for the static group was significantly higher compared with the average operation time for the articulating group. In addition, the average intraoperative blood loss level of the static group was significantly higher compared with the average blood loss in the articulating group. The average operation time for the static group was 119 minutes (range, 75-150 minutes), whereas the average intraoperative blood loss was 439 ml (range, 250-650 ml). The average operative time of the articulating group was 98 minutes (range, 65-135minutes), whereas the average intraoperative bleeding level was 358 ml (range, 150-600 ml). Four knees in the static group were administered with TTO due to stiff knee, whereas in the articular group, patients did not require additional TTO.

### 3.5. Knee Function

**Figure 6:**
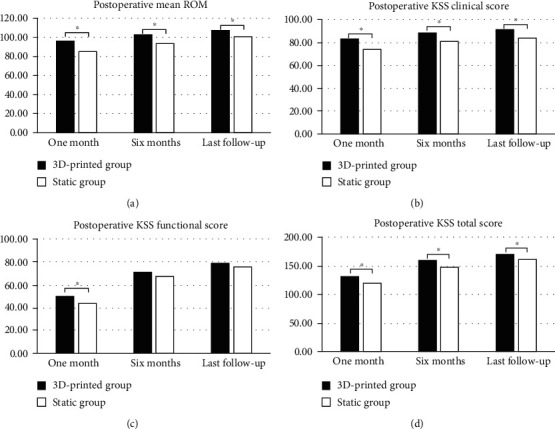
(a) Postoperative mean ROM, (b) Postoperative KSS clinical score, (c) postoperative KSS function score, and (d) postoperative KSS total score (^∗^*p* < 0.05).

The mean ROM of the knee joint was not significantly different between the two groups. A total of 5 patients received static spacer and severe knee movement limitation before operation. In addition, 4 patients received articular spacer. The number of patients who received static spacer and number of patients who received articular spacer was not significantly different. During the interim period, ROM of patients in the static group was almost 0 (0-8°), whereas the average ROM of articular group reached 88° (80°-100°). The average ROM of the static group after the second-stage revision was significantly different from the ROM of the articular group (*p* < 0.05). After the second-stage revision, average ROM in the static group was 80° (70° ~110°), whereas the average ROM in the articular group was 94° (80° ~115°). After a month and six months after surgery, and at the last follow-up, the joint range of motion in the articular group was significantly higher compared with the joint range of motion of the static group (Figure 6).

The KSS score of patients in the static group was not significantly different from KSS score of patients in the articular group during admission. KSS clinical score, functional score, and total score of patients in the articular group during the interim period were significantly higher compared with the scores in the static group (*p* < 0.05). Notably, KSS clinical score, functional score, and total score of patients in the static group during the interim period were 60 ± 6.3, 38 ± 3.3, and 98 ± 8.7, respectively, whereas the scores for the articular group were 75 ± 11.5, 42 ± 3.6, and 117 ± 14.4. Further, the KSS score after the final revision surgery in the articular was significantly higher compared with the KSS score of the articular group after the final revision surgery. The KSS score of the static group after the final revision surgery was 73 ± 5.8, 42 ± 4.6, and 115 ± 9.9, whereas the KSS score for the articular group after the final revision surgery was 82 ± 3.9, 49 ± 5.0, and 131 ± 8.9. Moreover, the articular group showed higher functional evaluation scores compared with functional evaluation scores for the static group at 1 month, 6 months, and during last follow-up. (Table 3).

### 3.6. Patient Satisfaction

Fifty-nine % (13/22) of patients in the fixation group reported overall satisfaction during the interim period, whereas in the joint group, ninety-five % (19/20) of patients reported overall satisfaction. The number of patients satisfied with overall treatment in the articular group was significantly higher compared with the number of satisfied patients in the static group (*p* < 0.05). After the final revision surgery, fifty-five % (12/22) of patients in the static group were satisfied with overall treatment, whereas 90% (18/20) of patients in the articular group were satisfied with overall treatment (Table 3).

## 4. Discussion

In this study, we designed a new articular spacer for two-staged PJI revision using 3D printing technology. Results from the retrospective study show that this new device reduces technical complexity of PJI surgery, improves surgical outcomes, and enhances quality of life of patients.

The recent studies have explored approaches to improve the design and formulation of bone cement spacer [22–24]. Currently, there are no reports on the clinical advantage of using static spacer over articular spacer [25, 26]. Previous studies reported that joint braking helps in preventing active infections; therefore, treatment of infected arthritis or even PJI should be adequately braked [27]. These findings led to the development and wide use of fixed joint. In addition, a variety of joint spacers are used in clinical practice to simulate the physiological function of the knee to achieve better quality of life. However, due to limitations in industrial advances, production and design of articulated spacers for PJI treatment are poor. Previous studies do not report advantages of articulated spacers over conventional spacers. In this study, we designed a novel articulated spacer using 3D printing technology which improved the quality of the product, functionality, and strength. In addition, the spacer can be customized to match the size of the bones of the patient. Furthermore, the findings of this study reveals that the novel articular spacer is more effective compared with the gold standard approach.

The infection cure rate of the novel articular spacer developed in this study was not significantly different compared with the cure rate of the fixed spacer. In addition to effective surgical debridement and rational antibiotic formulations, the design of the new spacer improved overall infection resistance compared with the fixed spacer. The morphology of the articulated prosthesis is designed to effectively fit and reduce the dead space in the joint (Figure 1). The cement spacer has a thicker carriage compared with the thin carriage design of metal prostheses; therefore, it improves overall strength and reduces volume of the suprapatellar pouch thus reducing joint effusion. The amount of antibiotic bone cement used in the preparation and installation of each articular spacer in this study was 50-60 g, which is above the 40 g used in the fixed spacer. Asians have a generally smaller body size; therefore, increase in dosage increases overall local antibiotic dosage for the fixed formula concentration. Moreover, the surface area of articular spacer is larger compared with that of static spacer of the same volume, facilitating effective release of antibiotics.

The spectrum of pathogens cultured in this study was similar to that reported in previous study, with high levels of *Staphylococcus aureus* observed [28, 29]. Improving the culture positivity rate of pathogenic bacteria and identifying causative bacteria are important in improving PJI treatment [30]. In the present study, culture-negative cases were observed, and a broad-spectrum antibiotic formulation was used for controlled release. These antibiotics are predominantly time-dependent, and some antibiotics have significant postantibiotic effects (e.g., vancomycin, meropenem, and fluconazole) [31, 32]. Rational administration of antibiotics is carried out to ensure prolonged dosage at minimum inhibitory concentrations. Previous study reports that amphotericin B is effective and is not easily affected by resistance in control of fungi and can be added to formulation of controlled release systems. However, amphotericin B is a concentration-dependent drug; therefore, it is mor effective when administered as a single high concentration dose.

X-ray analysis carried out in the interim period showed that patients using static spacer had more pronounced bone defects compared with patients using the articular spacer. Severe bone defects observed in the static spacer group may have been caused by limited knee motion and subsequent stress masking the contact area. Bone defects in the static group were mainly noninclusive defects occurring mainly on the tibial plateau side and the distal femur. Bone defects increase the cost of treatment and limit effectiveness of secondary surgical reconstruction.

The new articular spacer also provided favorable conditions for 2nd-stage revision. An articulated spacer-equipped limb maintained mobility and was less likely to develop stiff knee during the exclusion period. Therefore, surgical exposure, spacer removal, clean-up, and soft tissue balancing during the revision surgery are easier in articular spacers compared with static spacers. Patients using the new articular spacer showed less operative time and intraoperative bleeding for phase II revisions. In addition, none of the patients fitted with the articular spacer required a TTO osteotomy, which is technically difficult, time consuming, and is associated with poor prognosis. Previous studies reported that articulating spacer performs better compared with static spacer [33–35].

Previous studies report that the function scores of static spacer are not significantly different from function scores of articular spacer [13, 36, 37]. However, these findings do not report subjective feelings of patients. Use of a static spacer and fixing the knee in an extended position lead to loss of self-care abilities, such as wearing pants and using the toilet as the interim period takes more than two months. The overall satisfaction of patients using 3D printing-assisted articular spacers was higher compared with that of the control group. High overall satisfaction of patients can be attributed to higher early postoperative function scores and better joint function during the interim period.

Spacers designed in advance before surgery have a smoother friction interface, a more stable mechanical structure, and a better interim period joint function compared with hand-made bone cement spacers (static or articular spacers). However, spacers designed before surgery are few in the market. The model size of the mold of available commercial spacer types is limited. Traditional methods of manufacturing spacer molds are complicated and expensive, and it is difficult to customize products for a single patient. Use of 3D printing increases efficiency of spacer production and reduces the cost of production.

However, use of this technique has potential risks. There is no clinical evidence using in vivo studies to support long-term use of bone cement cast spacers. Long-term wear of the friction interface of PMMA material may loosen the articular spacer and cause instability of the joints. In addition, presence of worn particles is in the human body. In this study, the longest interim period was 4 months, and no visible particles or osteolysis symptoms were found at the 2nd revision. Secondly, use of self-made surgical tools should follow local medical laws and regulations, or approval from relevant authorities (such as FDA). Furthermore, this study had some limitations. Firstly, this is a retrospective study, and clinicians were not blinded to the type of spacer used when collecting data. Secondly, a low number of patients were recruited to the study. In addition, the length of follow-up time of the two groups was different. This is an unavoidable defect in a retrospective study comparing a new technique to a conventional one, but study shows knee functions seldomly change around 12 months after TKA surgery, and we assume that the functional outcomes are comparable at the time point we selected. These limitations may lead to potential bias in our results. Prospective randomized controlled trials with a larger number of cases and longer follow-up periods should be carried out to confirm effectiveness of articular spacers in PJI treatment.

## 5. Conclusion

This study reports preliminary findings of using 3D-printed articulating antibiotic cement spacer. The findings provide a new method for production of effective and personalized spacers. Articular spacer provides satisfactory range of motion during the interim period, prevents bone loss, facilitates second-stage reimplantation and postoperative rehabilitation, and results in low reinfection and complication rates. This technique could be considered to in a 2-stage revision surgery.

## Figures and Tables

**Table 1:** Patient baseline data and data during follow-up time.

	Gender (F/M)	Age (years)	Follow-up time (months)
Static group	7/15	67.2 ± 10.1	43 (30~61)
3D-printed group	8/12	65.5 ± 11.4	18 (8 ~ 28)

**Table 2:** Pathogen spectrum.

Organism	Spacer type	
	Static group	3D-printed articular group
MSSA	9	7
Staphylococcus epidermis	4	5
MRSA	2	1
Pseudomonas aeruginosa	2	1
Streptococcus	1	2
Aspergillus species	0	1
Other	2	1
No growth	2	2
Total	22	20

MSSA: methicillin susceptible *staphylococcus aureus*; MRSA: methicillin-resistant *staphylococcus aureus*.

**Table 3:** Comparison of outcomes between the static group and 3D-printed group.

	Static group (*n* = 22)	3D-printed articular group (*n* = 20)	*p* value
Interval time (wk)	13.1 (8-24)	12.8 (9-22)	0.75
Interim reinfection rate	1/22	1/20	0.95
Bone loss	22/22	4/20	0.00
Femoral bone loss(mm)	9.6 (5 ~ 20)	1.7 (0 ~ 10)	0.00
Tibial bone loss(mm)	5.5 (0 ~ 10)	1 (0 ~ 5)	0.00
TTO surgery	4/22	0/20	0.04
Second-stage operation time(min)	119 (75~150)	98 (65~135)	0.00
Operation bleeding volume	439 (250~650)	358 (150~600)	0.02
Preoperative KSS			
Clinical score	42 ± 5.7	40 ± 6.2	0.28
Function score	29 ± 5.7	28 ± 5.4	0.51
Total score	71 ± 10.5	68 ± 11	0.35
Interim KSS			
Clinical score	60 ± 6.3	75 ± 11.5	0.00
Function score	38 ± 3.3	42 ± 3.6	0.00
Total score	98 ± 8.7	117 ± 14.4	0.00
Postoperative KSS			
Clinical score	73 ± 5.8	82 ± 3.9	0.00
Function score	42 ± 4.6	49 ± 5.0	0.00
Total score	115 ± 9.9	131 ± 8.9	0.00
Mean ROM (degree)			
Preoperative ROM	71 (30~110)	73 (23~105)	0.74
Interim ROM	1 (0 ~ 8)	88 (80~100)	0.00
Postoperation ROM	80 (70~110)	94 (80~115)	0.00
Serious joint mobility disorder	5/22	4/20	0.83
Reinfection rate	1/22	1/20	0.95
Satisfaction rate	12/22	18/20	0.01

TTO: tibial tuberosity osteotomy; KSS: knee society score; ROM: range of motion.

## Data Availability

The data used to support the findings of this study are available from the corresponding author upon request.
