# Corrigendum to “Application of 3D Printing-Assisted Articulating Spacer in Two-Stage Revision Surgery for Periprosthetic Infection after Total Knee Arthroplasty: A Retrospective Observational Study”

**DOI:** 10.1155/2021/9792626

**Published:** 2021-05-31

**Authors:** Lingtong Kong, Jiawei Mei, Wufei Ge, Xiansheng Jin, Xiaoxuan Chen, Xianzuo Zhang, Chen Zhu

**Affiliations:** ^1^Department of Orthopedics, The Affiliated Provincial Hospital of Anhui Medical University, Hefei 230001, China; ^2^IAT-Chungu Joint Laboratory for Additive Manufacturing Anhui Chungu 3D Printing Institute of Intelligent Equipment and Industrial Technology, Wuhu 241200, China; ^3^College of Chemistry and Chemical Engineering, Xiamen University, Xiamen 361005, China; ^4^Department of Orthopedics, The First Affiliated Hospital of USTC, Division of Life Sciences and Medicine, University of Science and Technology of China, Hefei 230022, China

In the article titled “Application of 3D Printing-Assisted Articulating Spacer in Two-Stage Revision Surgery for Periprosthetic Infection after Total Knee Arthroplasty: A Retrospective Observational Study” [1], some of the authors were linked to the incorrect affiliations in the affiliation list. The correct author affiliations are now corrected in the author information above.
